# First person – Hartmut Cuny

**DOI:** 10.1242/dmm.049951

**Published:** 2022-11-14

## Abstract

First Person is a series of interviews with the first authors of a selection of papers published in Disease Models & Mechanisms, helping researchers promote themselves alongside their papers. Hartmut Cuny is first author on ‘ Maternal heterozygosity of *Slc6a19* causes metabolic perturbation and congenital NAD deficiency disorder in mice’, published in DMM. Hartmut is a senior postdoc in the lab of Prof. Sally Dunwoodie at Victor Chang Cardiac Research Institute, Sydney, Australia, investigating the genetic and environmental causes of congenital malformations.


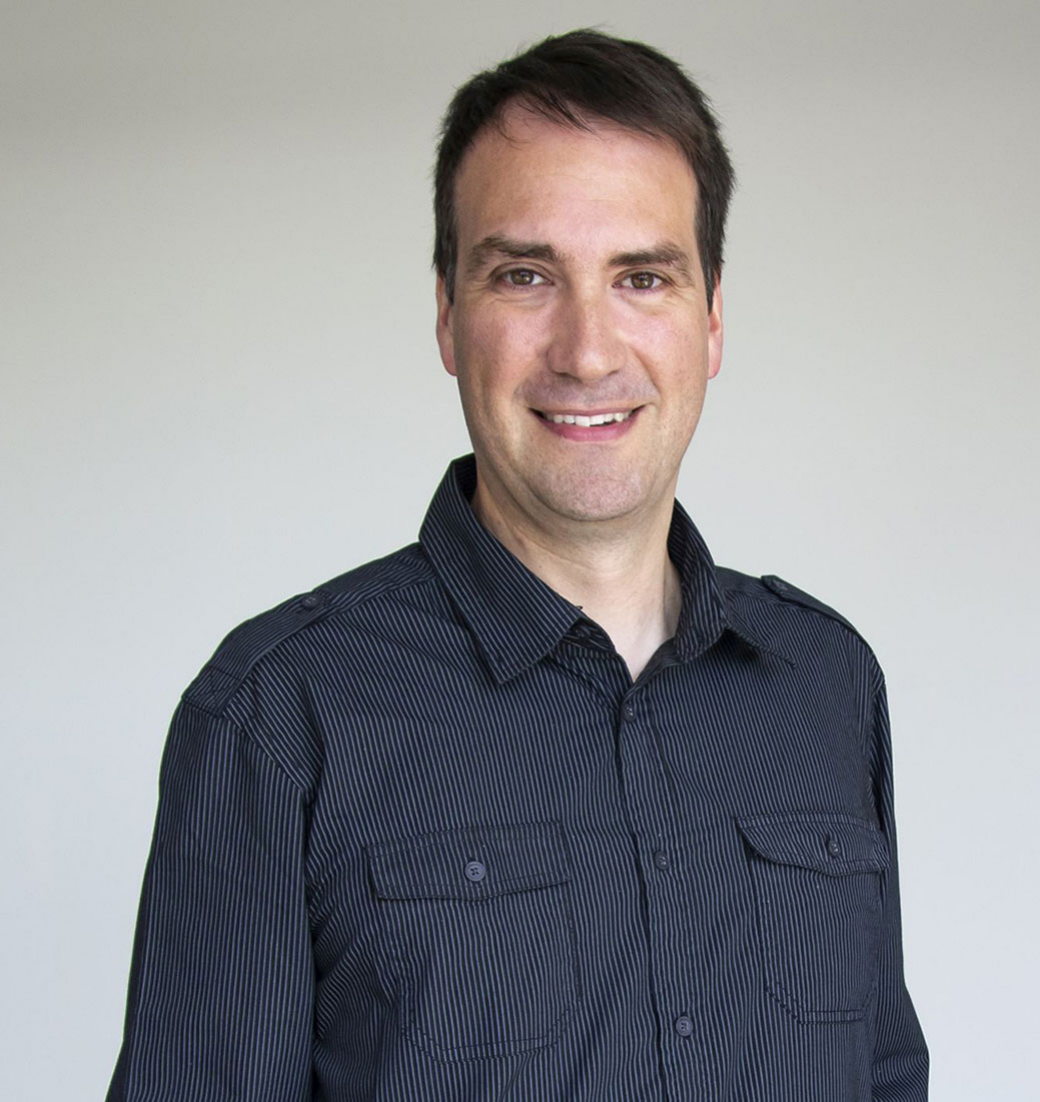


**Hartmut Cuny**

**How would you explain the main findings of your paper to non-scientific family and friends?**

Major congenital malformations (birth defects) are relatively common and a leading cause of death and disability in children worldwide. In many cases, the underlying causes of the birth defects are not known. A deficiency of nicotinamide adenine dinucleotide (NAD) during human pregnancy is one of the many known causes of birth defects, resulting in heart, kidney, spinal and other structural anomalies, and also leads to miscarriage. NAD is an important co-factor for numerous cellular functions in the body and generated from vitamin B3 or the essential amino acid tryptophan. Mouse models of NAD deficiency show the same adverse pregnancy outcomes as seen in humans, and these can be prevented by vitamin B3 during pregnancy.

Here, we showed that NAD deficiency and adverse pregnancy outcomes can also occur when the body's ability to absorb and retain tryptophan is affected. The gene we studied, *Slc6a19*, encodes the main transporter for tryptophan in the intestine and kidney. A mutation in one of its two copies in the genome (*Slc6a19* heterozygosity) by itself has no adverse health impact, but our mouse model showed that when this is combined with a diet reduced in tryptophan and vitamin B3, the mother's metabolism is perturbed. This reduces the amount of NAD that developing embryos require and results in malformation or miscarriage. Fortunately, these adverse pregnancy outcomes can be prevented if the mother receives sufficient vitamin B3, another precursor to generate NAD during pregnancy. In humans, loss-of-function mutations in the tryptophan transporter gene *SLC6A19* are fairly common, affecting roughly one in 600 people. Of these, women trying to conceive might be at risk of having a child with a birth defect or of miscarrying when their mutation in this tryptophan transporter coincides with other factors adversely affecting the generation of sufficient NAD in the body.

**What are the potential implications of these results for your field of research?**

In our current study, we showed that congenital NAD deficiency disorder (CNDD), a combination of congenital malformations caused by NAD deficiency, is not limited to cases with causative

“We showed that NAD deficiency and adverse pregnancy outcomes can occur when the body's ability to absorb and retain tryptophan is affected.”

biallelic loss-of-function variants in genes encoding enzymes that are directly involved in NAD synthesis. We showed that non-enzymatic components of NAD synthesis, as in our case the tryptophan transporter B^0^AT1 (encoded by *Slc6a19*), can be implicated in CNDD, too. Second, we showed in our mouse model that maternal heterozygous loss of function of *Slc6a19* by itself does not cause adverse pregnancy outcomes, but a gene–environment interaction between this genetic component and dietary NAD precursor restriction increased the incidence of CNDD compared to wild-type mothers. In humans, approximately one in 578 people carry a predicted loss-of-function variant in *SLC6A19*, according to the Genome Aggregation Database (gnomAD). Female carriers of such *SLC6A19* mutations might be predisposed to an adverse pregnancy outcome if there is insufficient tryptophan or vitamin B3 available to generate NAD during gestation. On a positive note, our mouse model in this study and our previous work showed that NAD deficiency can be overcome and CNDD prevented when the diet is supplemented with an NAD precursor that bypasses the genetic block, such as vitamin B3. So, there are possibilities to prevent CNDD if the underlying cause of the NAD deficiency is known.

**What are the main advantages and drawbacks of the experimental system you have used as it relates to the disease you are investigating?**

The main advantage of mouse models is that gene knockout lines can be generated relatively rapidly via the CRISPR/Cas9 gene-editing technology. This allows us to test models that replicate gene mutations found in patients. Furthermore, to investigate NAD precursor supply as an environmental factor, we use diets with defined composition that allow us to standardise the dietary supply of NAD precursors and directly monitor how levels of e.g. vitamin B3 or tryptophan affect pregnancy.

The metabolic pathways to synthesise and consume NAD are generally the same among mice and humans. But, a drawback of mice as a model system is that their metabolic rate is very different from that of humans because their bodies are so much smaller. Mice consume much more food relative to their body weight and can better compensate any genetic impairments of NAD synthesis than humans because of their disproportionately higher vitamin B3 intake. This means that humans are more sensitive towards perturbations of the NAD metabolism. So, any effect we see in mice, such as the gene–environment interaction between a heterozygous *Slc6a19* loss-of-function mutation and a diet reduced in NAD precursors, is likely to be even more impactful in humans.

**Figure:**
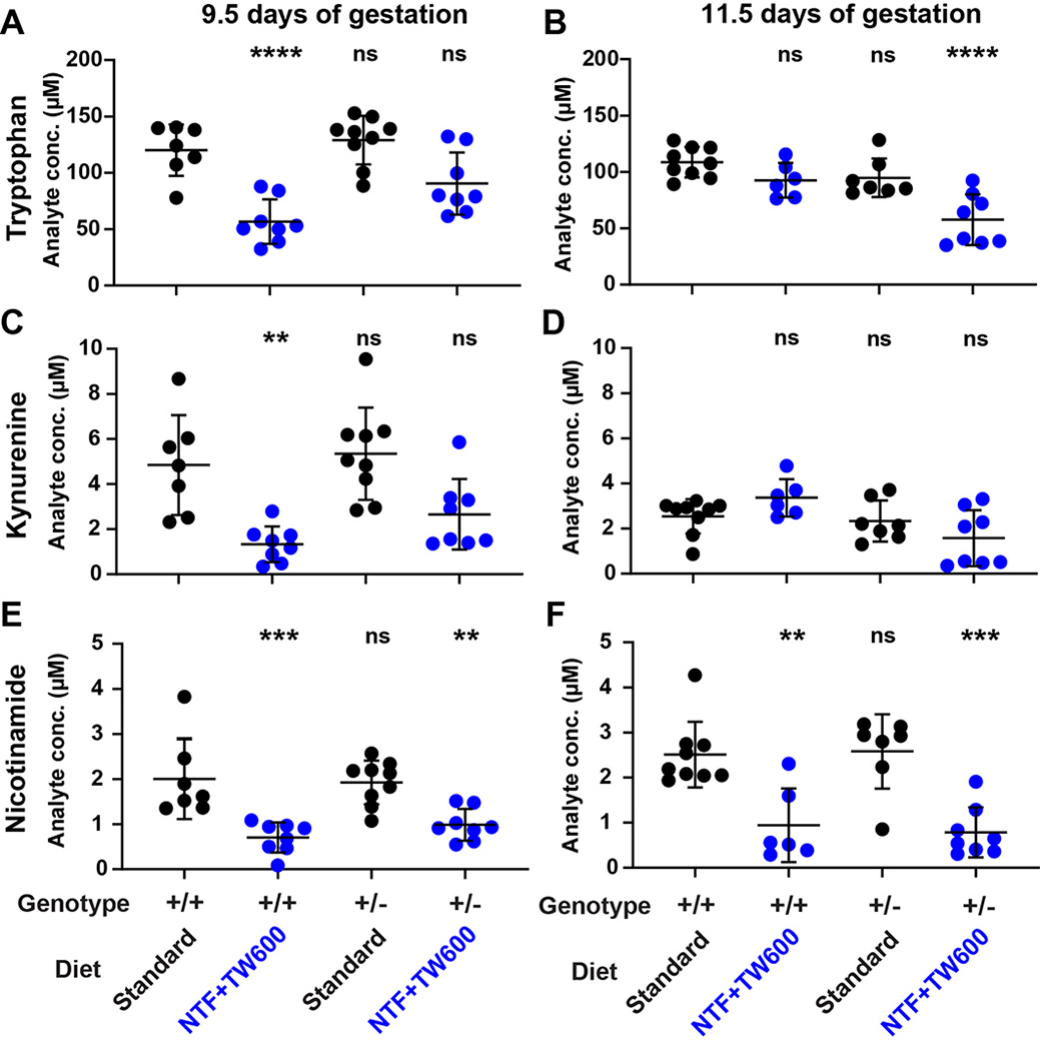
**Tryptophan, kynurenine and nicotinamide concentrations in plasma of pregnant wild-type and *Slc6a19*^+/−^ mice, measured by ultra-high-performance liquid chromatography–tandem mass spectrometry (UHPLC-MS/MS).** (A-F) Plasma metabolite concentrations were quantified at 9.5 (A,C,E) and 11.5 (B,D,F) days of gestation. The maternal *Slc6a19* genotype and diet are indicated at the bottom. Standard, standard diet; NTF+TW600, vitamin B3-depleted and tryptophan-restricted diet. Significance of differences in metabolite concentration relative to the wild-type standard diet group (first column in each graph) was assessed by one-way ANOVA with Tukey's multiple comparisons test (****P<0.0001, ***P<0.001, **P<0.01; ns, not significant).

**What has surprised you the most while conducting your research?**

What surprised me most were the intricate differences in how the maternal metabolism responded to the dietary and genetic factors. It was surprising to see that pregnant wild-type mice had lower levels of tryptophan and kynurenine (an intermediate of NAD synthesis produced from tryptophan) in their plasma than *Slc6a19* heterozygous mothers at embryonic day 9.5, but this was reversed 2 days later. We hypothesised that, due to compensatory mechanisms, the heterozygotes were initially better prepared for the change to the tryptophan-reduced diet. But, as the embryos grow, their demand for tryptophan increases. Two days later, the *Slc6a19* heterozygotes were no longer able to maintain a balance between using the tryptophan in the NAD synthesis pathway and providing enough tryptophan to their embryos for growth, evidenced by a significant decline in maternal NAD levels. In addition, *Slc6a19* heterozygous mothers on the precursor-restricted diet had significantly smaller embryos at late gestation compared to wild-type mothers. This was an unexpected finding, but, in light of our metabolic data, it makes sense.

**What do you think is the most significant challenge impacting your research at this time and how will this be addressed over the next 10 years?**

In recent years, our ability to sequence genomes, identify gene variants and predict their pathogenicity has improved dramatically. What remains as a bottleneck towards better understanding of disease causation is to link genetic variants to disease phenotypes. This requires that we understand the often-complex mechanisms by which a mutation causes the disease or alters a phenotype, the metabolism, etc. Such fundamental studies, starting with elucidating the biological function of a gene product and then understanding the molecular impact of a variant in the respective gene are time consuming and require suitable model systems. A major part of the challenge is that, in many cases, disease phenotypes are caused by multiple genetic and/or environmental factors. But each study linking a gene to a disease phenotype helps future studies and improves clinical diagnosis. I think we are going to see a stronger trend towards personalised medicine over the next 10 years, whereby a genetic diagnosis helps to guide patient treatment.

“A common issue in life sciences is limited resources and funding.”

**What changes do you think could improve the professional lives of scientists?**

A common issue in life sciences is limited resources and funding. It is important that fundamental research is funded well, too, because often this is where major research breakthroughs are happening which then directly benefit translational research and medicine. A related issue for early career scientists is that during the university studies they are usually well trained in becoming knowledgeable in their research subjects, but other skills and experience required in scientific research are not trained to the same extent. Career planning and mentoring should have more emphasis, also at later stages in the science career. On the other hand, regarding careers outside of academia, companies should generally be more open for scientists who e.g. just completed their university PhD or are otherwise transitioning from academia. Scientists are familiar with quickly adapting to new projects and ongoing learning/studying, so acquiring the desired job-specific skills is rarely a major burden.

**What's next for you?**

Another main project I have started working on is a clinical study to investigate what optimal levels of NAD in pregnancy are and what the prevalence of NAD deficiency among pregnant women is. In related side projects, we are collaborating with various clinicians who have patients with loss-of-function mutations in genes involved in NAD synthesis. It will be very exciting to apply and compare our findings from our mouse models to a human clinical context.

## References

[DMM049951C1] Cuny, H., Bozon, K., Kirk, R. B., Sheng, D. Z., Bröer, S. and Dunwoodie, S. L. (2022). Maternal heterozygosity of *Slc6a19* causes metabolic perturbation and congenital NAD deficiency disorder in mice. *Dis. Model. Mech.* 16, dmm049951. 10.1242/dmm.049951. 36374036 PMC9702539

